# Optimal dose of perineural dexmedetomidine to prolong analgesia after brachial plexus blockade: a systematic review and Meta-analysis of 57 randomized clinical trials

**DOI:** 10.1186/s12871-021-01452-0

**Published:** 2021-09-28

**Authors:** Hai Cai, Xing Fan, Pengjiu Feng, Xiaogang Wang, Yubo Xie

**Affiliations:** 1 Department of Anesthesiology, The First Affiliated Hospital of Guangxi Medical University, No. 6 Shuangyong Road, Nanning, 530021 Guangxi Zhuang Autonomous Region China; 2 Department of Anesthesiology, Liuzhou Hospital of Traditional Chinese Medicine, Liuzhou, Guangxi Zhuang Autonomous Region China

**Keywords:** Perineural dexmedetomidine, Adjuvant, Brachial plexus block, meta-analysis

## Abstract

**Background and Objectives:**

Peripheral injection of dexmedetomidine (DEX) has been widely used in regional anesthesia to prolong the duration of analgesia. However, the optimal perineural dose of DEX is still uncertain. It is important to elucidate this characteristic because DEX may cause dose-dependent complications. The aim of this meta-analysis was to determine the optimal dose of perineural DEX for prolonged analgesia after brachial plexus block (BPB) in adult patients undergoing upper limb surgery.

**Method:**

A search strategy was created to identify suitable randomized clinical trials (RCTs) in Embase, PubMed and The Cochrane Library from inception date to Jan, 2021. All adult patients undergoing upper limb surgery under BPB were eligible. The RCTs comparing DEX as an adjuvant to local anesthetic (LA) with LA alone for BPB were included. The primary outcome was duration of analgesia for perineural DEX. Secondary outcomes included visual analog scale (VAS) in 12 and 24 h, consumption of analgesics in 24 h, and adverse events.

**Results:**

Fifty-seven RCTs, including 3332 patients, were identified. The subgroup analyses and regression analyses revealed that perineural DEX dose of 30-50 μg is an appropriate dosage. With short−/intermediate-acting LAs, the mean difference (95% confidence interval [CI]) of analgesia duration with less than and more than 60 μg doses was 220.31 (153.13–287.48) minutes and 68.01 (36.37–99.66) minutes, respectively. With long-acting LAs, the mean differences (95% CI) with less than and more than 60 μg doses were 332.45 (288.43–376.48) minutes and 284.85 (220.31–349.39) minutes.

**Conclusion:**

30-50 μg DEX as adjuvant can provides a longer analgesic time compared to LA alone and it did not increase the risk of bradycardia and hypotension.

**Supplementary Information:**

The online version contains supplementary material available at 10.1186/s12871-021-01452-0.

## Introduction

Upper limb surgery is often performed under brachial plexus block (BPB), which is a series of regional anesthesia techniques and also contributes to reliable postoperative analgesia [1]. Single block and continuous catheter-based block are two different anesthesia regimens. Compared with continuous catheter-based block, more and more anesthesiologists prefer single block, because the catheter placement requires additional time, cost, and increases the risk of infection and neurological complications [2]. In order to prolong the time of single nerve block analgesia, more and more anesthesiologists add adjuvants to local anesthetics (LAs) [3]. Over the past decade, adjuvants of local anesthetics such as opioids [4], epinephrine [5], clonidine [6], magnesium [7], midazolam [8], dexamethasone [9], buprenorphine [10] and dexmedetomidine (DEX) [11] have been proved to prolong the analgesic time of nerve block, and have achieved varying degrees of success. Among these different kinds of adjuvants, DEX is more widely used. However, these adjuvants have different defects, such as the need for special equipment and monitoring, or the risk of complications that may delay discharge or lead to readmission [12].

Several prior meta-analyses [13–18] draw a conclusion that DEX is an effective perineural adjunct to LAs for producing prolonged analgesia duration. However, the use of DEX is not risk-free and may lead to complications in a dose-dependent manner, including hypertension, hypotension, bradycardia, excessive sedation, sleepiness, etc. It is vital to evaluate the optimal dose of perineural DEX that maximizes the analgesic benefit while minimizing associated perioperative risk. Since the publication of the previous meta-analysis, a large number of papers have been published focusing on different doses of peripheral DEX for BPB. The objective of current systematic review and meta-analysis was therefore to define the optimal dose of perineural DEX that prolongs analgesia after BPB in adult patients undergoing upper limb surgery.

## Materials and methods

This investigation followed the recommended process described in the “Preferred Reporting Items for Systematic Reviews and Meta-Analyses [19]” extension statement for reporting meta-analyses, and the protocol was registered on the International Platform of Registered Systematic Review and Meta-analysis Protocols (INPLASY; registration number: INPLASY202110066). A preliminary search suggested that vast majority of the published comparisons of interest have been conducted in the setting of BPB. Consequently, we decided to focus on the population of patients having upper limb surgery under BPB.

### Search strategy

Two authors (H Cai and X Fan) independently searched the electronic database including Embase, PubMed, and Cochrane Library from inception date to Jan, 2021. The search was restricted to articles in the English language. The online literature was searched using the following combination of medical subject heading terms and entry terms: “Brachial Plexus Block” or “Block, Brachial Plexus” or “Blocks, Brachial Plexus” or “Brachial Plexus Blocks” or “Brachial Plexus Anesthesia” or “Anesthesia, Brachial Plexus” or “Brachial Plexus Blockade” or “Blockade, Brachial Plexus” or “Blockades, Brachial Plexus” or “Brachial Plexus Blockades” or “Plexus Blockade, Brachial” or “Plexus Blockades, Brachial”. These search results were combined with “Dexmedetomidine” or “Dexmedetomidine Hydrochloride” or “MPV-1440” or “MPV1440” or “Precedex” or “MPV 1440” or “Hydrochloride, Dexmedetomidine”. We limited our search to title and abstract. Furthermore, the two authors (H Cai and X Fan) looked through the references of the relative papers to find additional studies.

### Including and excluding criteria

Studies were included if they met the following criteria: (1) only randomized clinical trials (RCTs); (2) comparison between perineural DEX with LA and only LA in single-injection BPB for upper limb surgery; (3) adult patients; and (4) in English.

Studies were excluded if they were (1) non-RCTs; (2) continuous or repeated nerve blocks; (3) DEX administered through non-perineural route or without LAs; (4) retracted articles; (5) Lack of relevant outcomes.

Four trials [20–23] investigated the effect of different dose of perineural DEX with LA by allocating patients into different separate groups were considered for the purpose of this meta-analysis. Trials [24–26] investigating the effect of perineural DEX with another perineural adjunct or without a placebo group, administering systemic DEX to all patients [27], or administering other α-2 agonist [28] than DEX were excluded.

### Assessment of methodological quality

Two reviewers (H Cai and P Feng) independently applied inclusion criteria from a review of the titles, abstracts, and keywords. Inconsistencies were settled by discussion or through consultation with the supervisor (Y Xie) until a consensus was reached. References were then searched by hand by the reviewer (H Cai and P Feng).

The reviewers (H Cai and P Feng) independently evaluated the methodological quality of the included RCTs according to the Cochrane Collaboration’s Risk of Bias Tool [29]. Studies were assessed for random sequence generation, allocation concealment, blinding of participants and personnel, blinding of outcome assessors, incomplete outcome data, selective reporting, and any other potential source of bias. The results of every trial were used following consensus between the 2 reviewers. Inconsistencies were settled by discussion or through consultation with the superior reviewer (Y Xie) until a consensus was reached.

### Data extraction and outcome assessment

Two reviewers (H Cai and X Wang) independently extracted the data from articles including first author, publication year, sample size, nerve localization techniques, perineural DEX dosage or dosages per average body weight, LA concentration and volume, and types. If they disagreed with each other, disagreements were either discussed to reach a consensus between the 2 reviewers or decided by superior (Y Xie). The source study text and tables were used to extract means, standard deviations (SDs), number of events, and total number of participants. If the trials just provided graphs, we extract data using GetData Graph Digitizer software [30]. The median and interquartile range were used for mean and SD approximations as follows: the mean was estimated as equivalent to the median and the SD was approximated to be the interquartile range divided by 1.35 or the 95% CI range divided by 4 [31]. All opioids were converted into equianalgesic doses of intravenous (IV) morphine for analysis (IV morphine 10 mg = oral morphine 30 mg = IV hydromorphone 1.5 mg = oral hydromorphone 7.5 mg = IV pethidine 75 mg = oral oxycodone 20 mg = IV tramadol 100 mg = intramuscular diclofenac 100 mg) [32]. Pain scores reported as visual, verbal, or numeric rating scales were converted to a standardized 0–10 analog scale for quantitative evaluations.

The primary outcome was duration of analgesia, defined as the time interval between block performance or onset time of sensory blockade and the time of first analgesic request or initial pain report [33]. The secondary outcomes included VAS in 12 and 24 h postoperatively, cumulative IV morphine consumption at 24 h postoperatively, and adverse events such as bradycardia and hypotension.

### Statistical analysis

One reviewer (H Cai) input the data and another (X Fan) checked its accuracy. Meta-analysis was implemented using Review Manager software (RevMan for Windows, version 5.4, Cochrane Collaboration, Oxford, UK). We estimated the mean differences for continuous data and risk difference for categorical data between groups, with an overall estimate of the pooled effect. The *χ*^2^ test was used for heterogeneity analysis, and heterogeneity was assessed by *I*^2^. If *I*^2^ < 50%, the fixed effects model was used; if *I*^2^ ≥ 50%, the random effects model was used and the heterogeneity was assessed [15]. Our primary outcome, duration of analgesia, was analyzed according to the dose of perineural DEX injected for each type of LA (short−/intermediate-acting LAs and long-acting LAs). We further undertook an exploratory analysis for each type of LAs in an attempt to account for heterogeneity and grouped trials by DEX dosage group (low doses: ≤ 60 μg; moderate doses: > 60 μg), by BPB localization (interscalene, supraclavicular, infraclavicular, axillary) and by regional anesthetic technique (anatomic landmarks, nerve stimulation, ultrasound). Finally, the relationship between dose of perineural DEX and mean increase in duration of analgesia was investigated for each type of local anesthetic with a regression analyses using the JMP 13 statistical package (SAS Institute, Cary, NC) [32]. The likelihood of publication bias was assessed by drawing a funnel plot of standard error of the mean difference (y-axis) as a function of the mean difference (x-axis) of our primary outcome [33]. This assessment was performed using STATA software (STATA for Windows, version 16.0, Stata Corp, Texas, USA). Results are presented as the mean difference or risk difference with 95% CI. A 2-sided *P* value < 0.05 was considered significant.

## Results

### Search results

**Fig. 1:**
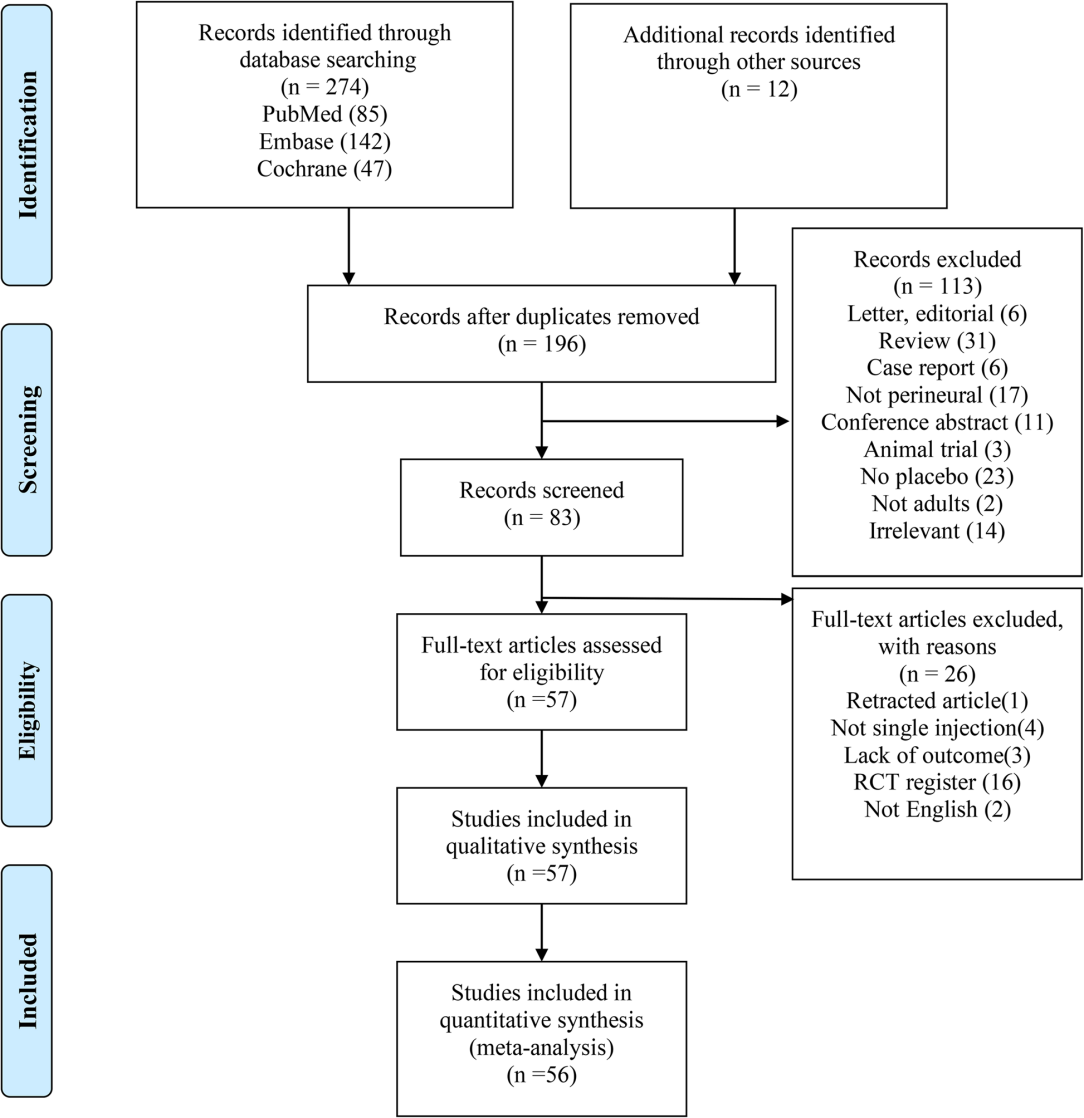
Preferred Reporting Items for Systematic Reviews and Meta-Analyses flow diagram summarizing included and excluded randomized controlled trials

Of the 286 trials identified from the literature search strategy and other sources, 57 RCTs [20–23, 34–86] met the inclusion criteria, representing a total of 3332 patients. Among the 286 articles, 90 duplicate articles were excluded initially. Then, 113 articles were excluded after screened titles and abstracts. 26 articles were excluded after full-text reading for the following reasons: retracted article, not single injection, lack of required outcomes, RCT registration, not English. Finally, 57 RCTs remained eligible to meet the inclusion criteria for the current meta-analysis. And the flow diagram of study selection is shown in (Fig. 1).

### Study characteristics

A detailed description of all the included studies is shown in (Table 1). All of the included studies were published between the years 2010 and 2020. The vast majority of the studies were conducted at international centers in Asia. Across all included studies, a total of 3332 patients were assessed. DEX was used as an adjuvant to several different local anesthetics, which included ropivacaine [23, 34, 39, 41, 42, 45–47, 49, 50, 52, 56, 57, 59, 63–66, 68, 72–74, 76, 78, 80, 81, 84, 85], bupivacaine [20, 35, 37, 38, 40, 51, 53–55, 67, 69, 71], levobupivacaine [21, 43, 44, 48, 60–62, 79, 83], and lidocaine [22, 36, 70, 77, 86]. Across the studies, the dose of DEX ranged from 0.5 μg/kg to a total of 150 μg. Local anesthetic dosages also varied across the studies.

**Table 1:** Characteristics of including trials

Author	Year	Country	Groups(n)	CON of LA-Total volume	DEX dose	Weight (kg)	Block/location	Technique	Outcomes
Hwang	2020	Korea	1.Ropivacaine + DEX (25) 2.Ropivacaine + NS (25)	0.75%-9 ml	100 μg	68.50 ± 13.20	Interscalene	Ultrasound	DOA, VAS
Nicholas	2020	India	1.Ropivacaine + DEX (27) 2.Ropivacaine + NS (27)	0.5%-21 ml	0.5 μg/kg	68.89	Axillary	Ultrasound	AE
Shahtaheri	2020	Iran	1.Lidocaine + DEX (33) 2.Lidocaine + NS (33)	1.5%-35 ml	0.5 μg/kg	NR	Axillary	Nerve stimulator	OC, VAS
Singh	2020	India	1.Ropivacaine + DEX (20) 2.Ropivacaine + NS (20)	0.5%-30 ml	1 μg/kg	67.50 ± 9.30	Supraclavicular	Ultrasound	DOA, OC, AE
Avula	2019	India	1.Bupivacaine + DEX (30) 2.Bupivacaine + NS (30)	0.5%-20.75 ml	75 μg	68.83 ± 5.38	Supraclavicular	Ultrasound	DOA, AE
Hassan	2019	Egypt	1.Bupivacaine + Lidocaine + DEX (15) 2.Bupivacaine + Lidocaine + NS (15)	0.5–2%-25 ml	100 μg	NR	Supraclavicular	Ultrasound	DOA
Nazir	2019	India	1.Ropivacaine + DEX (30) 2.Ropivacaine + NS (30)	0.5%-30 ml	50 μg	64.88 ± 6.70	Supraclavicular	Nerve stimulator	DOA
Sharma	2019	Nepal	1.Ropivacaine + DEX (30) 2.Ropivacaine + NS (30)	0.5%-31 ml	0.75 μg/kg	64.30 ± 5.90	Supraclavicular	Ultrasound	DOA, AE
Singh	2019	India	1.Ropivacaine + DEX (30) 2.Ropivacaine + NS (30)	0.5%-31 ml	100 μg	53.33 ± 8.21	Supraclavicular	Nerve stimulator	DOA, AE
Yaghoobi	2019	Iran	1.Lidocaine + DEX (25) 2.Lidocaine + NS (25)	2%-30 ml	1 μg/kg	NR	Infraclavicular	Ultrasound	DOA
Akhondzadeh	2018	Iran	1.Lidocaine + DEX (36) 2.Lidocaine + NS (31)	2%-30 ml	1 μg/kg	68.72 ± 7.87	Supraclavicular	Ultrasound	DOA, VAS, OC
Elyazed	2018	Egypt	1.Ropivacaine + DEX (35) 2.Ropivacaine + NS (35)	0.5%-39 ml	100 μg	76.50 ± 8.90	Infraclavicular	Ultrasound	DOA, VAS, OC, AE
Hamed	2018	Egypt	1.Bupivacaine + DEX (20) 2.Bupivacaine + NS (20)	1.5 mg/kg-40 ml	1 μg/kg	72.80 ± 7.30	Supraclavicular	Ultrasound	DOA, AE
He	2018	China	1.Ropivacaine + DEX (28) 2.Ropivacaine + NS (28)	0.375%-40 ml	1 μg/kg	77.32 ± 14.18	Coracoid approach	Nerve stimulator	DOA, VAS, OC
Jung	2018	Korea	1.Levobupivacaine + DEX (25) 2.Levobupivacaine + DEX (25) 3.Levobupivacaine + DEX (24) 4.Levobupivacaine + NS (23)	0.5%-22 ml	1 μg/kg 1.5 μg/kg 2 μg/kg	69.37 ± 11.33 63.81 ± 9.44 69.80 ± 16.85	Interscalene	Ultrasound	DOA, VAS, OC
Kaur	2018	India	1.Levobupivacaine + DEX (40) 2.Levobupivacaine + NS (40)	0.5%-30 ml	1 μg/kg	NR	Supraclavicular	Ultrasound	DOA, AE
Koraki	2018	Greece	1.Ropivacaine + DEX (19) 2.Ropivacaine + NS (18)	0.5%-16 ml	100 μg	NR	Axillary	Ultrasound	DOA, AE
Liu	2018	China	1.Ropivacaine + DEX (57) 2.Ropivacaine + NS (57)	0.375%-20 ml	100 μg	NR	NR	Nerve stimulator	DOA, VAS, AE
Mangal	2018	India	1.Ropivacaine + DEX (44) 2.Ropivacaine + NS (43)	0.75%-22 ml	1 μg/kg	60.36 ± 6.41	Supraclavicular	Ultrasound	DOA, OC, AE
Mathew	2018	India	1.Ropivacaine + DEX (20) 2.Ropivacaine + NS (20)	0.5%-30 ml	1 μg/kg	68.35 ± 11.70	Supraclavicular	Ultrasound	DOA
Pillai	2018	India	1.Bupivacaine + DEX (33) 2.Bupivacaine + DEX (33) 3.Bupivacaine + NS (33)	0.5%-27 ml	20 μg 40 μg	63.52 ± 14.14 66.64 ± 13.62	Supraclavicular	Ultrasound	DOA
Aksu	2017	Turkey	1.Bupivacaine + DEX (25) 2.Bupivacaine + NS (25)	0.33%-15 ml	1 μg/kg	76.40 ± 10.80	Supraclavicular	Ultrasound, Nerve stimulator	DOA, AE
Bisui	2017	India	1.Levobupivacaine + DEX (34) 2.Levobupivacaine + NS (33)	0.5%-30 ml	0.75 μg/kg	59.00 ± 7.64	Supraclavicular	Nerve stimulator	DOA, VAS
Chinnappa	2017	UAE	1.Ropivacaine + DEX (30) 2.Ropivacaine + NS (30)	0.5%-31 ml	1 μg/kg	64.90 ± 11.30	Supraclavicular	Nerve stimulator	DOA, AE
Farooq	2017	India	1.Ropivacaine + DEX (35) 2.Ropivacaine + NS (35)	0.75%-35 ml	1 μg/kg	50.90 ± 10.20	Interscalene	Nerve stimulator	DOA
Rashmi	2017	India	1.Ropivacaine + DEX (30) 2.Ropivacaine + NS (30)	0.75%-30.5 ml	50 μg	61.00 ± 12.16	Interscalene	Nerve stimulator	DOA
Thakur	2017	India	1.Lidocaine + DEX (30) + adrenaline 2.Lidocaine + DEX (30) + adrenaline 3.Lidocaine + NS (30) + adrenaline	2%-30 ml	1 μg/kg 0.5 μg/kg	50.77 ± 10.64 48.37 ± 10.48	Axillary	Landmark	DOA
Wang	2017	China	1.Ropivacaine + DEX (31) 2.Ropivacaine + NS (27)	0.5%-25 ml	0.75 μg/kg	61.00 ± 5.00	Interscalene	Nerve stimulator	AE
Abdallah	2016	Canada	1.Ropivacaine + DEX (33) 2.Ropivacaine + NS (32)	0.5%-16 ml	0.5 μg/kg	82.3	Interscalene	Ultrasound	DOA, OC, AE, VAS
Arun	2016	India	1.Ropivacaine + DEX (30) 2.Ropivacaine + NS (30)	0.75%-26 ml	50 μg	68.57 ± 2.00	Axillary	Nerve stimulator	DOA, AE
Bangera	2016	India	1.Ropivacaine + DEX (40) 2.Ropivacaine + NS (40)	0.375%-40 ml	1 μg/kg	60.33 ± 7.11	Axillary	Nerve stimulator	DOA, AE
Lee	2016	Korea	1.Ropivacaine + DEX (17) 2.Ropivacaine + NS (17)	0.5%-22 ml	100 μg	65.6 ± 4.80	Axillary	Ultrasound, Nerve stimulator	AE
Nazir	2016	India	1.Bupivacaine + DEX (35) 2.Bupivacaine + NS (35)	0.25%-40 ml	1 μg/kg	52.00 ± 8.70	Supraclavicular	Ultrasound	DOA
Singh	2016	India	1.Levobupivacaine + DEX (29) 2.Levobupivacaine + NS (28)	0.5%-31 ml	100 μg	NR	Supraclavicular	Nerve stimulator	DOA, AE
Tandon	2016	India	1.Levobupivacaine + DEX (30) 2.Levobupivacaine + NS (30)	0.5%-31 ml	100 μg	63.10 ± 4.28	Supraclavicular	Nerve stimulator	DOA, AE
Bharti	2015	India	1.Ropivacaine + lidocaine + DEX (27) + adrenaline 2.Ropivacaine + lidocaine + NS (27) + adrenaline	0.75–2%-0.5 ml/kg	1 μg/kg	62.52 ± 9.22	Supraclavicular	Ultrasound	DOA, AE
Gurajala	2015	India	1.Ropivacaine + DEX (16) 2.Ropivacaine + NS (15)	0.5%-35 ml	50 μg	62.13 ± 13.61	Supraclavicular	Nerve stimulator	DOA, AE
Karthik	2015	India	1.Levobupivacaine + DEX (50) 2.Levobupivacaine + NS (50)	0.5%-40 ml	1 μg/kg	66.42 ± 5.34	Axillary	Landmark	AE
Kathuria	2015	India	1.Ropivacaine + DEX (20) 2.Ropivacaine + NS (20)	0.5%-30 ml	50 μg	72.00 ± 11.67	Supraclavicular	Ultrasound	DOA, OC
Kaur	2015	India	1.Levobupivacaine+lidocaine+DEX (50) 2.Levobupivacaine+lidocaine+NS (50)	0.25–1%-40 ml	1 μg/kg	66.18 ± 7.32	Supraclavicular	Nerve stimulator	DOA
Manohar	2015	India	1.Bupivacaine + DEX (30) 2.Bupivacaine + NS (30)	0.5%-30 ml	50 μg	53.26 ± 10.49	Supraclavicular	Nerve stimulator	DOA, AE
Tiwari	2015	India	1.Ropivacaine + DEX (60) 2.Ropivacaine + NS (60)	0.75%-20 ml	50 μg	60.27 ± 9.11	Supraclavicular	Ultrasound	DOA, AE
Agarwal	2014	India	1.Bupivacaine + DEX (25) 2.Bupivacaine + NS (25)	0.325%-31 ml	100 μg	64.00 ± 9.40	Supraclavicular	Nerve stimulator	DOA, AE
Biswas	2014	India	1.Levobupivacaine + DEX (30) 2.Levobupivacaine + NS (30)	0.5%-36 ml	100 μg	71.36 ± 9.38	Supraclavicular	Nerve stimulator	DOA
Fritsch	2014	Austria	1.Ropivacaine + DEX (16) 2.Ropivacaine + NS (15)	0.5%-12 ml	150 μg	NR	Interscalene	Ultrasound	VAS
Megha	2014	India	1.Bupivacaine + lidocaine + DEX (20) 2.Bupivacaine + lidocaine + NS (20)	0.5–2%-30 ml	50 μg	NR	Supraclavicular	Nerve stimulator	DOA, AE
Mirkheshti	2014	Iran	1.Lidocaine + DEX (34) 2.Lidocaine + NS (34)	1.5%-30 ml	100 μg	72.00 ± 9.00	Infraclavicular	Ultrasound	DOA
Nema	2014	India	1.Ropivacaine + DEX (30) 2.Ropivacaine + NS (30)	0.75%-30 ml	50 μg	NR	Supraclavicular	Landmark	DOA
Song	2014	Korea	1.Mepivacaine + DEX(10) 2.Mepivacaine + NS(10)	1%-40 ml	1 μg/kg	64.80 ± 9.60	Infraclavicular	Nerve stimulator	DOA
Zhang	2014	China	1.Ropivacaine + DEX (15) 2.Ropivacaine + DEX (15) 3.Ropivacaine + NS (15)	0.33%-41 ml	100 μg 50 μg	66.40 ± 7.85 65.47 ± 12.14	Axillary	Nerve stimulator	AE
Dar	2013	India	1.Ropivacaine + DEX (40) 2.Ropivacaine + NS (40)	0.5%-41 ml	50 μg	72.20 ± 9.01	Axillary	Nerve stimulator	DOA, AE
Patki	2013	India	1.Ropivacaine + DEX (30) 2.Ropivacaine + NS (30)	0.5%-30.5 ml	50 μg	NR	Supraclavicular	Landmark	DOA
Ammar	2012	Egypt	1.Bupivacaine + DEX (30) 2.Bupivacaine + NS (30)	0.33%-30 ml	0.75 μg/kg	80.50 ± 9.50	Infraclavicular	Ultrasound	DOA, OC
Gandhi	2012	India	1.Bupivacaine + DEX (35) 2.Bupivacaine + NS (35)	0.25%-40 ml	30 μg	51.40 ± 10.60	Supraclavicular	Landmark	DOA, AE
Hanoura	2012	Egypt	1.Bupivacaine + DEX (25) 2.Bupivacaine + NS (25)	0.25%-41 ml	100 μg	NR	Axillary	Nerve stimulator	DOA, VAS
Kaygusuz	2012	Turkey	1.Levobupivacaine + DEX (30) 2.Levobupivacaine + NS (30)	0.5%-40 ml	1 μg/kg	73.75 ± 8.11	Axillary	Nerve stimulator	DOA
Esmaoglu	2010	Turkey	1.Levobupivacaine + DEX (30) 2.Levobupivacaine + NS (30)	0.5%-41 ml	100 μg	72.20 ± 9.01	Axillary	Nerve stimulator	DOA, AE

Abbreviations: CON, concentration; LA, local anesthetics; DEX, dexmedetomidine; kg, kilogram; NS, normal saline; ml, milliliter; μg, microgram; DOA, duration of analgesia; VAS, visual analogue scale; OC, Opioid consumption; AE, adverse event; NR, not reported

### Risk-of-bias assessment of included studies

**Fig. 2:**
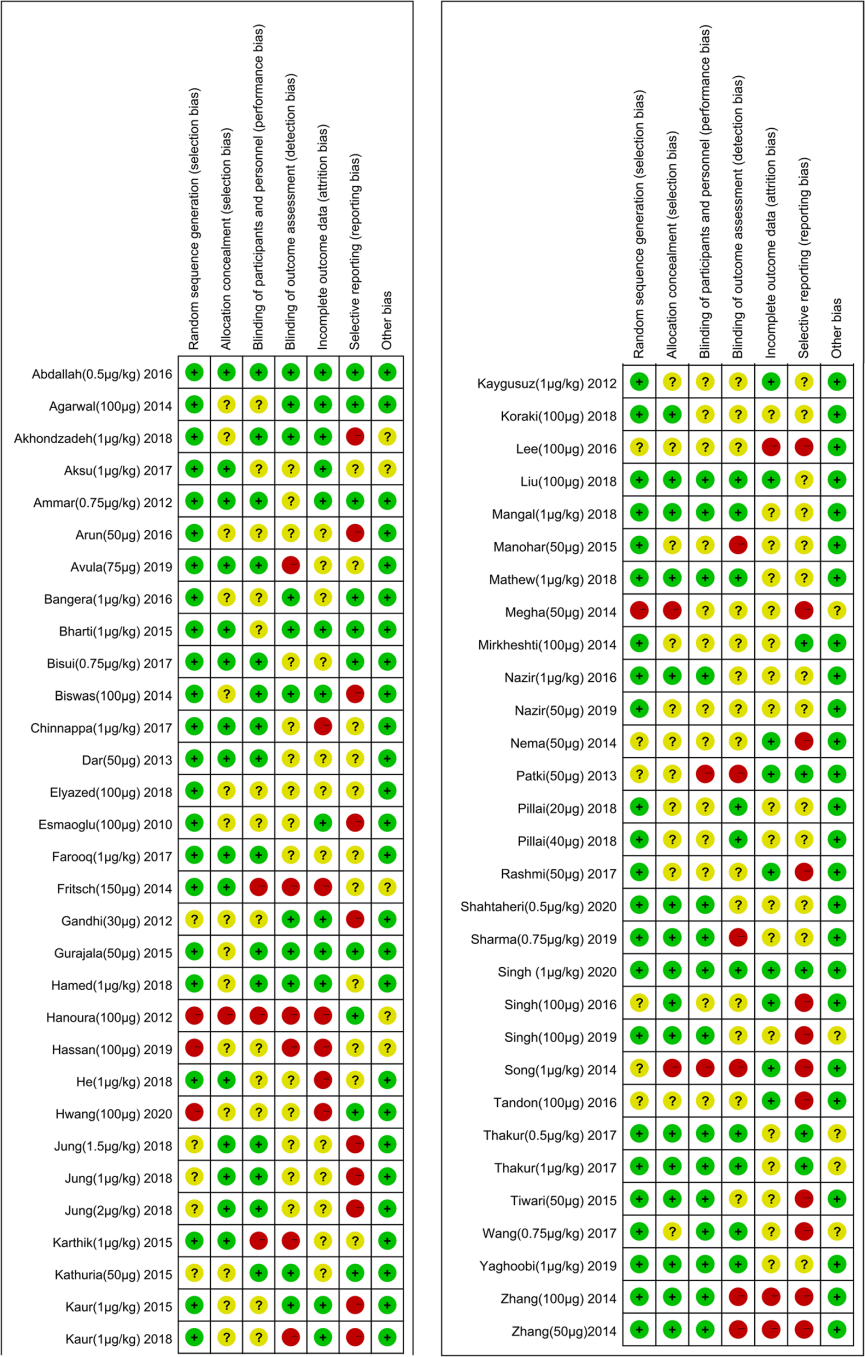
Risk of bias summary. Review authors’ judgements about each risk of bias item for each included study. Green circle, low risk of bias; orange circle, high risk of bias; yellow circle, unclear risk of bias

Two independent reviewers (H Cai and P Feng) assessed the risk-of-bias of all included studies. The vast majority of the studies had an unclear risk of bias due to the lack of sufficient methodological reporting. Several studies were classified as high risk of bias for allocation concealment due to the lack of clarity in methods used. A full risk-of-bias summary for all included studies is shown in (Fig. 2). Visual inspection of the funnel plot for primary outcomes suggests obviously publication bias.

### Duration of analgesia

**Fig. 3:**
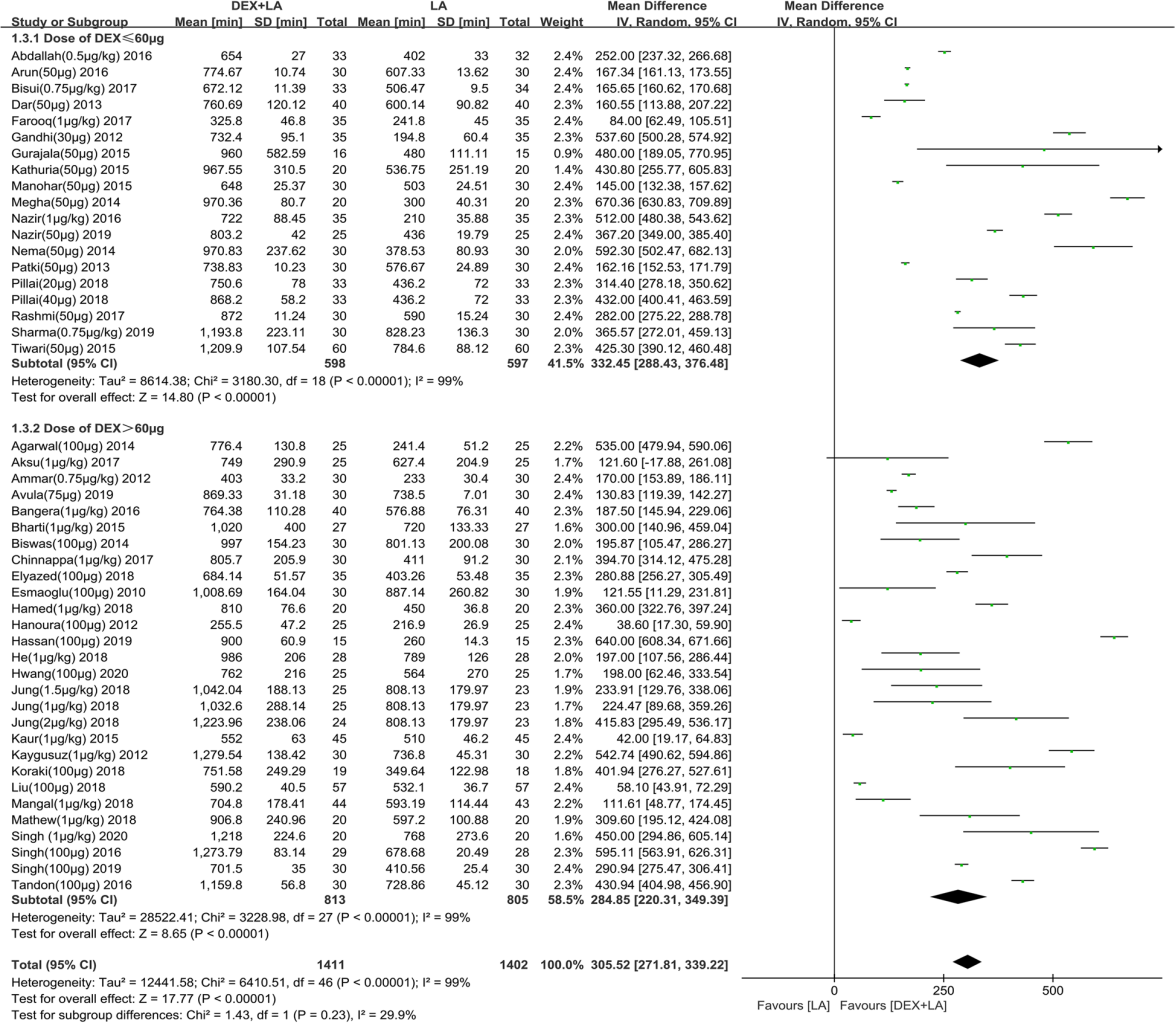
Effect of perineural DEX by dose administered (≤60 μg or>60 μg) on DOA when combined with long-acting LA. Abbreviations: DEX, dexmedetomidine; CI, confidence interval; DOA, duration of analgesia; LA, local anesthetic; IV, intravenous

**Fig. 4:**
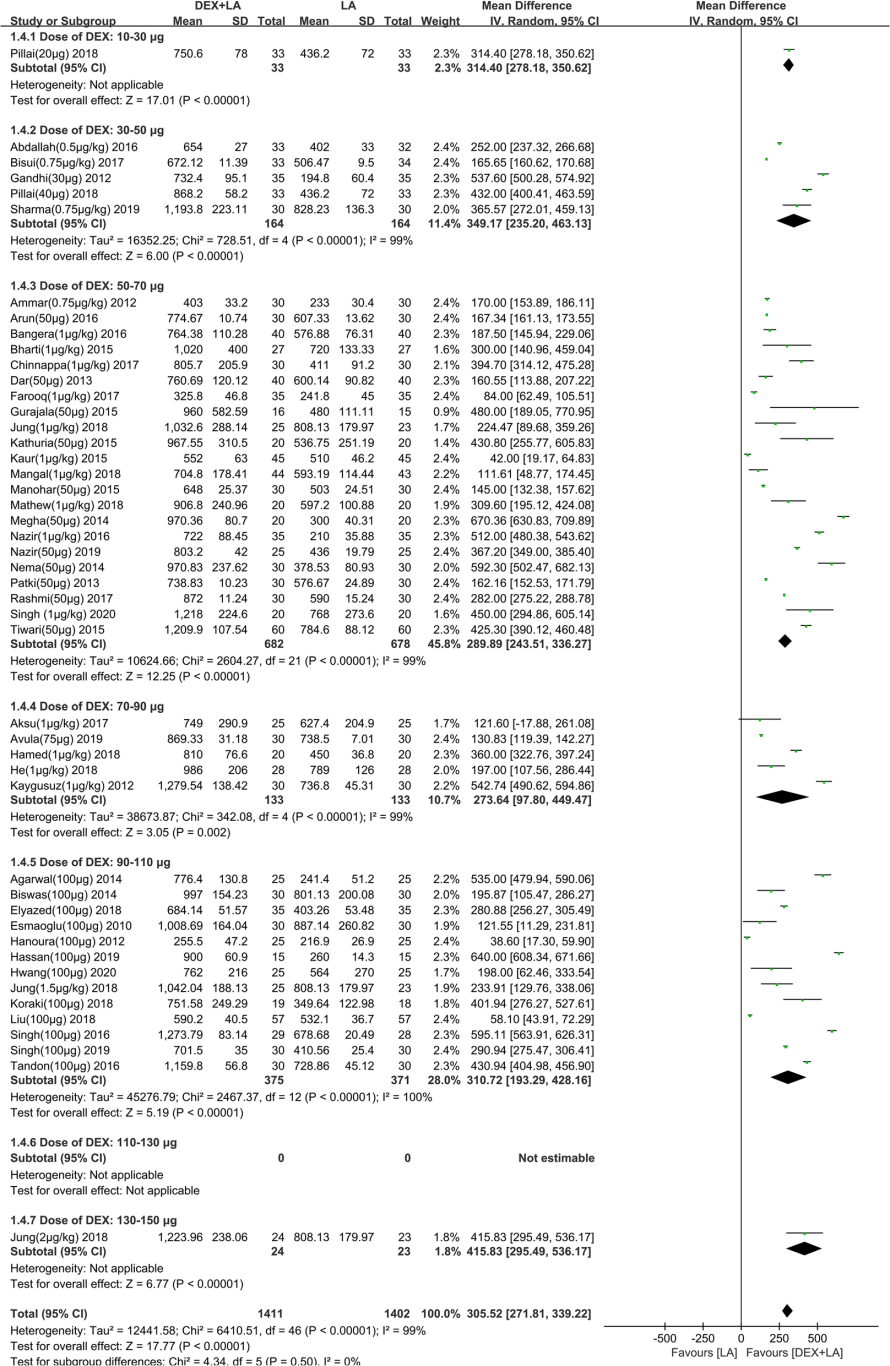
Subgroup analysis by 20 μg increments of perineural DEX on DOA when combined with long-acting LA. Abbreviations: DEX, dexmedetomidine; CI, confidence interval; DOA, duration of analgesia; LA, local anesthetic; IV, intravenous

The duration of analgesia was assessed by 50 studies [20–22, 34–49, 51–57, 59–63, 65–72, 75, 76, 78–84, 86], all of them (*n* = 3218) had sufficient information to allow for pooling. With short−/intermediate-acting LAs, the mean difference (95% confidence interval [CI]) of duration of analgesia with ≤60 μg and >60 μg DEX were 220.31 (153.13 to 287.48) minutes and 68.01 (36.37 to 99.66) minutes, respectively (test for subgroup difference: *P*<0.0001) (Additional file 1). The forest plot for subgroup analysis of short−/intermediate-acting LAs by dose group was not available because of the lack of sufficient data. With long-acting LAs, the mean difference (95% CI) of duration of analgesia with ≤60 μg and>60 μg DEX were 332.45 (288.43 to 376.48) minutes and 284.85 (220.31 to 349.39) minutes, respectively (test for subgroup difference: *P* = 0.23) (Fig. 3). The forest plot for subgroup analysis of long-acting LAs by different dose group indicated that 30-50 μg DEX as adjuvant could prolong the duration of analgesia by 349.17 min compared with LA alone (95% CI: 235.20 to 463.13 min) (Fig. 4). With the obvious heterogeneity the subgroup analysis was conducted according to types of BPB approaches and location technology (Additional file 2). Unfortunately, we still did not find the source of heterogeneity. Regression analysis showed that the mean line and fitting line overlapped, and basically in the horizontal position when combined with long-acting LAs (*R*^2^ = 0.001408; *P*<0.0001) (Additional file 3). However, when combined with short−/intermediate-acting LAs, regression analysis showed that the angle between the mean line and the fitting line is large (*R*^2^ = 0.55371; *P* = 0.0465) (Additional file 4). The above indicated that DEX as LA adjuvants on BPB significantly prolonged the duration of analgesia. Subgroup analysis and regression analysis showed that 30-50 μg DEX could prolong the duration of analgesia up to about 5 h.

### Pain-related outcome

Ten studies [21, 44, 47, 50, 54, 56, 57, 65, 77, 80] evaluated the pain score at 12 h postoperatively, and nine studies [21, 34, 47, 50, 56, 57, 65, 77, 80] for 24 h. It was found that the Pain score at rest at 12 h postoperatively was significantly reduced with perineural DEX. However, the pain score at rest at 24 h postoperatively was not statistically significant. Meanwhile, seven studies [21, 34, 36, 38, 47, 56, 59] accessed the anesthetic consumption in 24 h after surgery. It shows that DEX, no matter less than or more than 60 μg, can reduce the consumption of IV morphine in 24 h after operation. In conclusion, DEX as adjuvant can reduce postoperative pain score in 12 h and reduce the consumption of postoperative analgesics (Table 2).

**Table 2:** Pain-related outcomes (Abbreviations: RCT, randomized Clinical Trial; DEX, dexmedetomidine; LA, local anesthetic; CI, confidence interval)

Outcome	RCT	DEX + LA		LA		Mean Difference (95% CI)	***I***^**2**^(%)	***P*** Value for Overall Effect	***P*** Value for Subgroup Differences	Quality of Evidence (GRADE)
		Mean (SD)	N	Mean (SD)	N					
**Pain score at rest at 12 h postoperatively (analog scale, 0–10)**										
DEX ≤ 60 μg	Bisui (0.75 μg/kg) 2017	4.27(0.45)	33	5.76(0.65)	34	−1.43(−1.88 to −0.99)	8	<0.00001		
	Shahtaheri (0.5 μg/kg) 2020	0.50(4.76)	33	1.02(2.33)	33					
DEX>60 μg	Elyazed (100 μg) 2018	1.90(1.00)	35	2.86(0.60)	35	− 1.63(−2.07 to − 1.20)	76	<0.00001		
	Fritsch (150 μg) 2014	0.23(0.97)	16	2.19(1.76)	15					
	Hanoura (100 μg) 2012	3.70(1.20)	25	4.50(1.40)	25					
	He (1 μg/kg) 2018	0.93(0.98)	28	1.93(1.15)	28					
	Hwang (100 μg) 2020	4.20(2.50)	25	6.10(2.20)	25					
	Jung (1.5 μg/kg) 2018	0.61(0.99)	25	2.80(1.40)	23					
	Jung (1 μg/kg) 2018	0.52(0.79)	25	2.80(1.40)	23					
	Jung (2 μg/kg) 2018	0.00(0.00)	24	2.80(1.40)	23					
	Liu (100 μg) 2018	2.20(0.90)	57	4.20(1.10)	57					
	Singh (1 μg/kg) 2020	0.00(0.74)	20	2.00(2.04)	20					
total						−1.57(−1.91 to −1.23)	71	<0.00001	0.53	Very low
**Pain score at rest at 24 h postoperatively (analog scale, 0–10)**										
DEX ≤ 60 μg	Abdallah (0.5 μg/kg) 2016	5.50(0.45)	33	5.60(0.45)	32	−0.10(−0.32 to 0.12)	0	0.38		
	Shahtaheri (0.5 μg/kg) 2020	0.97(3.24)	33	0.90(5.58)	33					
DEX>60 μg	Elyazed (100 μg) 2018	1.60(0.50)	35	1.80(0.60)	35	−0.71(−1.93 to 0.52)	98	0.26		
	Fritsch (150 μg) 2014	2.68(2.93)	16	2.10(2.20)	15					
	He (1 μg/kg) 2018	1.54(0.84)	28	4.36(1.31)	28					
	Hwang (100 μg) 2020	3.30(1.30)	25	3.70(1.50)	25					
	Jung (1.5 μg/kg) 2018	5.13(1.15)	25	4.35(1.01)	23					
	Jung (1 μg/kg) 2018	4.10(1.04)	25	4.35(1.01)	23					
	Jung (2 μg/kg) 2018	4.90(1.20)	24	4.35(1.01)	23					
	Liu (100 μg) 2018	2.10(0.40)	57	5.40(0.80)	57					
	Singh (1 μg/kg) 2020	0.00(0.74)	20	1.00(1.48)	20					
total						−0.60(−1.61 to 0.42)	98	0.25	0.34	Very low
**Cumulative IV morphine consumption at 24 h postoperatively (mg)**										
DEX ≤ 60 μg	Abdallah (0.5 μg/kg) 2016	21.3(0.87)	33	27.3(1.16)	32	−6.01(−6.50 to −5.52)	0	<0.00001		
	Kathuria (50 μg) 2015	5.63(3.33)	20	12.0(5.66)	20					
DEX>60 μg	Akhondzadeh (1 μg/kg) 2018	12.04(6.74)	36	26.62(7.58)	36	−5.03(−8.52 to −1.11)	96	0.01		
	Ammar (0.75 μg/kg) 2012	4.90(5.93)	30	13.6(8.89)	30					
	Elyazed (100 μg) 2018	1.67(3.12)	35	7.31(1.6)	35					
	He (1 μg/kg) 2018	7.30(4.40)	28	15.9(7.60)	28					
	Jung (1.5 μg/kg) 2018	8.00(3.49)	25	6.5(3.38)	23					
	Jung (1 μg/kg) 2018	7.00(3.16)	25	6.5(3.38)	23					
	Jung (2 μg/kg) 2018	6.92(2.53)	24	6.5(3.38)	23					
total						−5.03(−7.54 to −2.51)	95	<0.0001	0.53	Very low

### DEX-related adverse event

**Fig. 5:**
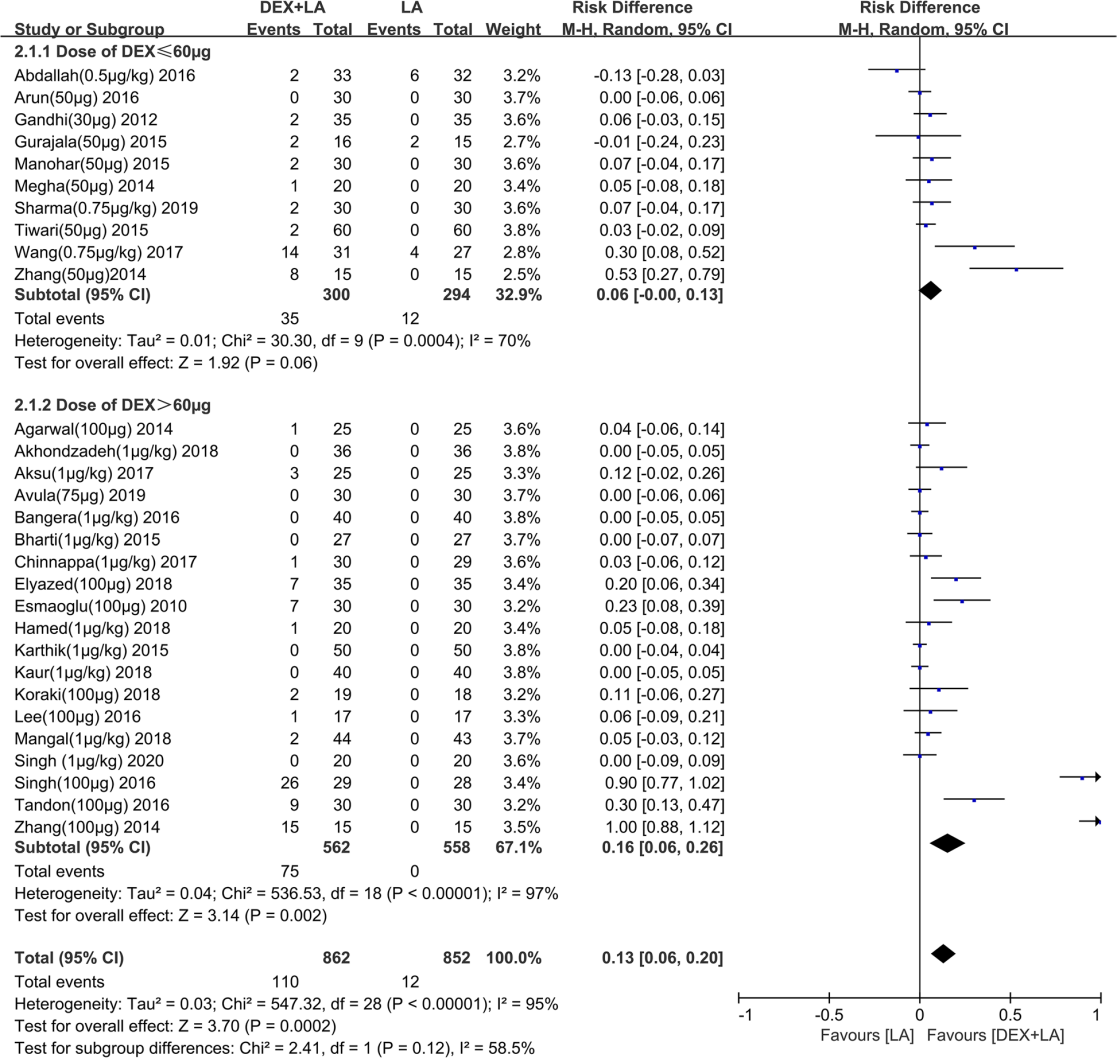
Effect of perineural DEX by dose administered (≤60 μg or>60 μg) on bradycardia when combined with long-acting LA. Abbreviations: DEX, dexmedetomidine; CI, confidence interval; LA, local anesthetic; IV, intravenous

The incidence of bradycardia and hypotension was described in 28 studies [23, 34–37, 39–42, 45, 47, 48, 51–53, 58, 61, 63, 64, 66, 67, 69, 78–80, 83–85] and 26 studies [23, 34–36, 39–42, 45–48, 51–53, 58, 63, 64, 66, 67, 69, 78–80, 84, 85] respectively. Pooled analysis showed that perineural DEX>60 μg increased the risk of bradycardia (risk difference [RD]: 0.16, 95% CI: 0.06 to 0.26, *I*^2^ = 97%, *P* = 0.002) (Fig. 5) in comparison to control, and this result was robust to sensitivity analysis by eliminating two [23, 79] notable outliers (RD: 0.05, 95% CI: 0.01 to 0.05, *I*^2^ = 73%, *P* = 0.01) (Additional file 5). Nevertheless, perineural DEX ≤ 60 μg did not increase the risk of bradycardia (RD: 0.06, 95% CI: − 0.00 to 0.13, *I*^2^ = 70%, *P* = 0.06) (Fig. 5) when comparing to control, and this result was also robust to sensitivity analysis by eliminating two [23, 85] notable outliers (RD: 0.03, 95% CI: − 0.00 to 0.06, *I*^2^ = 0%, *P* = 0.09) (Additional file 5). With regard to hypotension, the meta-analysis concluded that DEX>60 μg as adjuvant obviously increased the risk of it (RD: 0.07, 95% CI: 0.01 to 0.13, *I*^2^ = 90%, *P* = 0. 02) (Additional file 6). However, perineural DEX ≤ 60 μg did not increased the risk of hypotension (RD: 0.01, 95% CI: − 0.01 to 0.04, *I*^2^ = 13%, *P* = 0.34) (Additional file 6). Overall, peripheral DEX>60 μg increases the risk of adverse events, such as bradycardia and hypotension.

### Publication bias

**Fig. 6:**
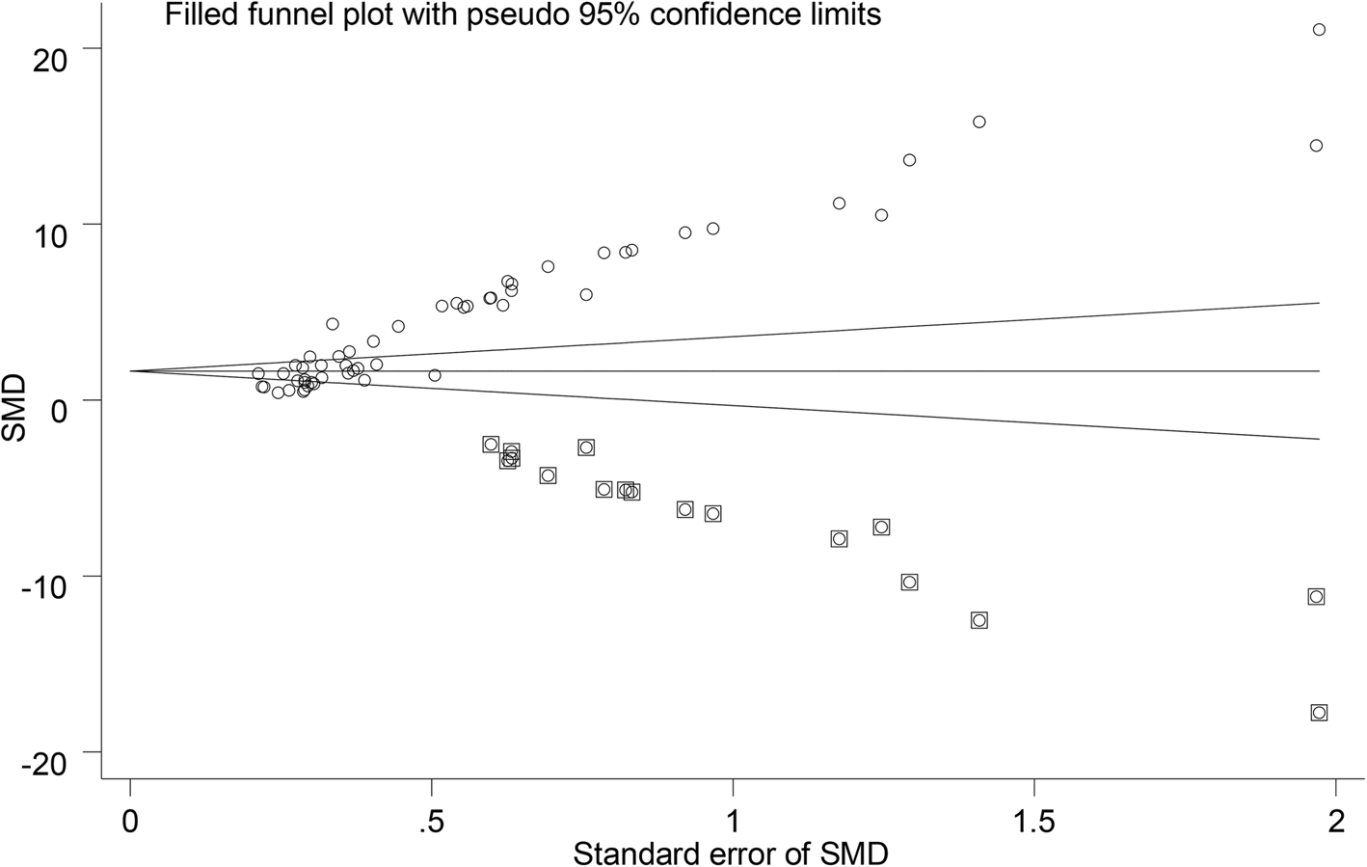
Trim and fill test. It showed significant publication bias in the primary outcome (duration of analgesia) (*P* = 0.00). Abbreviations: SMD, standardized mean difference

With regard to the funnel plot for our primary outcome, the Duval and Tweedie’s trim and fill test showed the standardized mean difference for the combined studies to be 4.20 (95% CI: 3.63 to 4.78), suggesting that 17 studies are missing (Fig. 6). We rated the quality of evidence for each outcome following the Grades of Recommendation, Assessment, Development, and Evaluation (GRADE) Working Group system [87] (Table 3).

**Table 3:** Summary of findings

Quality assessment							Summary of findings			
Outcome	Limitations	Inconsistency	Indirectness	Imprecision	Publication Bias	Number of Patients in DEX Group	Number of Patients in Control Group	Mean Difference or Relative Risk (95% CI)	P Value for Overall Effect	Quality of Evidence (GRADE)
DOA when combined with short−/intermediate-acting LA (min)	Concealment not clear in most studies	Serious inconsistency	Moderate indirectness	No serious imprecision	7 studies missing for our primary outcome	140	135	126.01(44.22 to 207.81)	**0.003**	Very low quality
DOA when combined with long-acting LA (min)	Concealment not clear in most studies	Serious inconsistency	Moderate indirectness	No serious imprecision	7 studies missing for our primary outcome	1411	1402	305.52(271.81 to 339.22)	**<0.00001**	Very low quality
Pain score at rest at 12 h postoperatively (analog scale, 0–10)	Concealment not clear in most studies	Serious inconsistency	No serious indirectness	Moderate imprecision	7 studies missing for our primary outcome	346	341	−1.57(−1.91 to −1.23)	**<0.00001**	Very low quality
Pain score at rest at 24 h postoperatively (analog scale, 0–10)	Concealment not clear in most studies	Serious inconsistency	No serious indirectness	Moderate imprecision	7 studies missing for our primary outcome	321	314	−0.60(−1.61 to 0.42)	0.25	Very low quality
Cumulative IV morphine consumption at 24 h postoperatively (mg)	Concealment not clear in most studies	Serious inconsistency	No serious indirectness	Moderate imprecision	7 studies missing for our primary outcome	256	250	−5.03(−7.54 to −2.51)	**<0.0001**	Very low quality
Rate of bradycardia	28 of 57 trials reported that outcome	Serious inconsistency	No serious indirectness	Serious imprecision	7 studies missing for our primary outcome	862	852	0.13(0.06 to 0.20)	**0.0002**	Very low quality
Rate of hypotension	26 of 57 trials reported that outcome	Serious inconsistency	No serious indirectness	Serious imprecision	7 studies missing for our primary outcome	802	789	0.05(0.01 to 0.09)	**0.007**	Very low quality

Abbreviations: DEX, dexmedetomidine; CI, confidence interval; GRADE, Grades of Recommendation, Assessment, Development, and Evaluation; DOA, duration of analgesia; LA, local anesthetic; IV, intravenous

## Discussion

This systematic review and meta-analysis explored the optimal dose of DEX as an adjuvant to prolong the duration of analgesia after BPB in adult patients undergoing upper limb surgery. Based on 58 RCTs, including a total of 3332 patients, our subgroup analysis and regression analysis suggest that 30-50 μg of DEX as an adjuvant represents an optimal dose and prolongs analgesia by 5 h, when combined with long-acting local anesthetics; higher doses may lead to DEX-related adverse events such as bradycardia and hypotension.

The first meta-analysis focused on DEX as an adjuvant, published in 2013 [13], indicated that there are presently insufficient safety data to support the use of perineural DEX in the clinical setting. Four years later, in 2017, the same team in an updated meta-analysis [16] confirmed that using perineural DEX improves BPB onset, quality, and analgesia. After that, four other meta-analysis [14, 15, 17, 18] further confirmed the efficacy of DEX as adjuvant. One of the them found that DEX, particularly at doses greater than 50 μg, holds a great potential for clinicians wishing to quicken the onset and prolong the duration of anesthesia [14]. In our meta-analysis, the interaction between dose of perineural DEX and mean increase in duration of analgesia was explored by grouping every 20 micrograms of DEX. Regression analysis was used to predict the relationship between them. Finally, we come to our conclusion.

The quality of evidence for our primary outcome was rated as very low due to the lack of clear allocation concealment, high coefficient of heterogeneity, absence of consistent definition of the primary outcome and significant publication bias. This means that we have little confidence in the effect estimation, and the real effect is likely to be very different from the effect estimation.

Our review comes with several strengths and potential limitations. Firstly, ours is the first review to pool a large number of RCTs on this topic and provide greater insights into the optimal dose of DEX. While the prior review [18] in 2018 just included 12 RCTs, we were able to include an additional 45. Secondly, there was a high consistency in the evaluation of each parameter in this meta-analysis. Finally, we successfully analyzed the influencing factors of DEX on duration of analgesia, including different doses, BPB approaches and positioning techniques; however, since these factors were not randomized in the included studies, there was an inherent risk of bias in this analysis.

It is worth noting that one of the limitations of our review is the high heterogeneity of primary outcome. Furthermore, even subgroup analysis could not successfully solve the problem of heterogeneity attributed to the smaller sample sizes of individual studies, the potential variation in the study populations, and the different methods that could have been used to measure the outcomes in question. Secondly, most of included trials were performed in developing countries and published in non-anesthesia journals. This may also be the reason for the high heterogeneity.

## Conclusion

In conclusion, there is very low quality evidence that 30-50 μg of perineural DEX represents an appropriate dosage, which prolongs analgesia duration by a mean period of 5 h when combined with long-acting LAs. Perineural DEX above 60 μg can significantly increase the incidence of adverse events such as bradycardia or hypotension. More high-quality methodological and strictly defined RCTs are urgently needed to further evaluate the advantages and disadvantages of DEX as an adjuvant.

## Supplementary Information

**Additional file 1.** Effect of perineural DEX by dose administered (≤60 μg or>60 μg) on DOA when combined with short−/intermediate-acting. Abbreviations: DEX, dexmedetomidine; CI, confidence interval; DOA, duration of analgesia; LA, local anesthetic; IV, intravenous.

**Additional file 2.** Subgroup analyses of DEX on DOA by BPB approaches and localization techniques. Abbreviations: DEX, dexmedetomidine; DOA, duration of analgesia; BPB, brachial plexus block; MD, mean difference; CI, confidence interval; LA, local anesthetic.

**Additional file 3.** Regression analysis of perineural DEX dose and mean increase in DOA when combined with long-acting LAs (pink line: mean line; green line: fitting line). Abbreviations: DEX, dexmedetomidine; DOA, duration of analgesia; LA, local anesthetic.

**Additional file 4.** Regression analysis of perineural DEX dose and mean increase in DOA when combined with short−/intermediate-acting LAs (pink line: mean line; green line: fitting line). Abbreviations: DEX, dexmedetomidine; DOA, duration of analgesia; LA, local anesthetic.

**Additional file 5.** Sensitivity analysis by eliminating two notable outliers respectively. Abbreviations: DEX, dexmedetomidine; LA, local anesthetic; CI, confidence interval.

**Additional file 6.** Effect of perineural DEX by dose administered (≤60 μg or>60 μg) on hypotension. Abbreviations: DEX, dexmedetomidine; LA, local anesthetic; CI, confidence interval.

## Data Availability

All data generated or analyzed during this study are included in this published article.

## Abbreviations

LA: local anesthetic
BPB: brachial plexus block
RCTs: randomized clinical trials
CI: confidence interval
DEX: dexmedetomidine
RD: risk difference
MD: mean difference
