# Supplementary material for: Optimal dose of perineural dexmedetomidine to prolong analgesia after brachial plexus blockade: a systematic review and Meta-analysis of 57 randomized clinical trials
Source: BMC Anesthesiol. 2021 Sep 28;21:233. doi: 10.1186/s12871-021-01452-0 (PMC8477554; doi:10.1186/s12871-021-01452-0)
Supplement: Supplementary file 1 — Additional file 1. Effect of perineural DEX by dose administered (≤60 μg or>60 μg) on DOA when combined with short−/intermediate-acting. Abbreviations: DEX, dexmedetomidine; CI, confidence interval; DOA, duration of analgesia; LA, local anesthetic; IV, intravenous. [file 12871_2021_1452_MOESM1_ESM.docx]

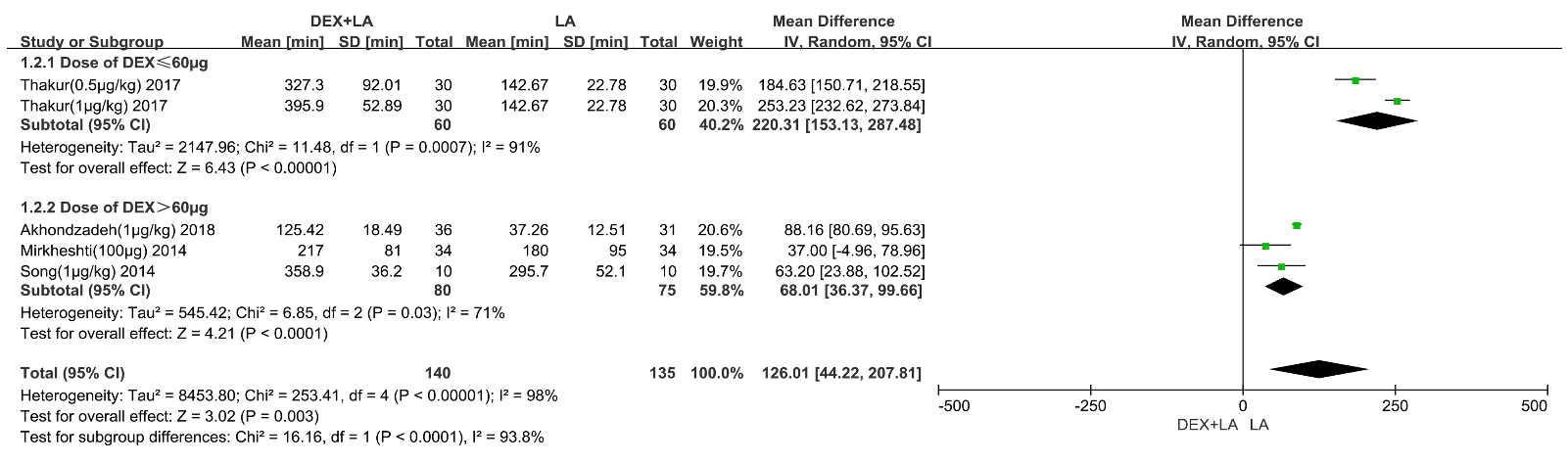


**Additional file 1: Figure S1** Effect of perineural DEX by dose administered (≤60μg or＞60μg) on DOA when combined with short-/intermediate-acting. Abbreviations: DEX, dexmedetomidine; CI, confidence interval; DOA, duration of analgesia; LA, local anesthetic; IV, intravenous
