# Supplementary material for: Optimal dose of perineural dexmedetomidine to prolong analgesia after brachial plexus blockade: a systematic review and Meta-analysis of 57 randomized clinical trials
Source: BMC Anesthesiol. 2021 Sep 28;21:233. doi: 10.1186/s12871-021-01452-0 (PMC8477554; doi:10.1186/s12871-021-01452-0)
Supplement: Supplementary file 2 — Additional file 2. Subgroup analyses of DEX on DOA by BPB approaches and localization techniques. Abbreviations: DEX, dexmedetomidine; DOA, duration of analgesia; BPB, brachial plexus block; MD, mean difference; CI, confidence interval; LA, local anesthetic. [file 12871_2021_1452_MOESM2_ESM.docx]

**Additional file 2: Table S1** Subgroup analyses of DEX on DOA by BPB approaches and localization techniques.

|  |  | No. of studies (total patients) | MD (min) | 95% CI (min) | *I*^2^ | *p* |
| --- | --- | --- | --- | --- | --- | --- |
| **short-/intermediate-acting LA** |  | 6(325) | 111.87 | 40.29 to 183.46 | 98 | **0.002** |
| BPB approaches | Axillary | 2(120) | 220.31 | 153.13 to 287.48 | 91 | **＜0.00001** |
|  | Infraclavicular | 3(138) | 46.93 | 23.87 to 70.00 | 0 | **＜0.0001** |
|  | Supraclavicular | 1(67) | 88.16 | 80.69 to 95.63 | - | **＜0.00001** |
| Localization techniques | Landmark | 2(120) | 220.31 | 153.13 to 287.48 | 91 | **＜0.00001** |
|  | Nerve stimulator | 1(20) | 63.20 | 23.88 to 102.52 | - | **0.002** |
|  | Ultrasound | 3(185) | 58.89 | 18.87 to 98.92 | 82 | **0.004** |
| **long-acting LA** |  | 47(2779) | 305.77 | 298.39 to 401.60 | 99 | **＜0.00001** |
| BPB approaches | Axillary | 7(427) | 226.96 | 138.27 to 315.64 | 98 | **＜0.00001** |
|  | Coracoid approach | 1(56) | 197.00 | 107.56 to 286.44 | - | **＜0.0001** |
|  | Infraclavicular | 2(130) | 225.03 | 116.38 to 333.69 | 98 | **＜0.0001** |
|  | Interscalene | 7(388) | 235.55 | 166.66 to 304.43 | 98 | **＜0.00001** |
|  | Supraclavicular | 30(1778) | 349.99 | 298.39 to 401.60 | 99 | **＜0.00001** |
| Localization techniques | Landmark | 3(190) | 429.01 | 122.21 to 735.81 | 100 | **0.006** |
|  | Nerve stimulator | 23(1425) | 268.55 | 223.09 to 314.01 | 99 | **＜0.00001** |
|  | Ultrasound | 23(1328) | 310.19 | 245.21 to 375.18 | 99 | **＜0.00001** |

Abbreviations: DEX, dexmedetomidine; DOA, duration of analgesia; BPB, brachial plexus block; MD, mean difference; CI, confidence interval; LA, local anesthetic
