# Supplementary material for: Optimal dose of perineural dexmedetomidine to prolong analgesia after brachial plexus blockade: a systematic review and Meta-analysis of 57 randomized clinical trials
Source: BMC Anesthesiol. 2021 Sep 28;21:233. doi: 10.1186/s12871-021-01452-0 (PMC8477554; doi:10.1186/s12871-021-01452-0)
Supplement: Supplementary file 3 — Additional file 3. Regression analysis of perineural DEX dose and mean increase in DOA when combined with long-acting LAs (pink line: mean line; green line: fitting line). Abbreviations: DEX, dexmedetomidine; DOA, duration of analgesia; LA, local anesthetic. [file 12871_2021_1452_MOESM3_ESM.docx]

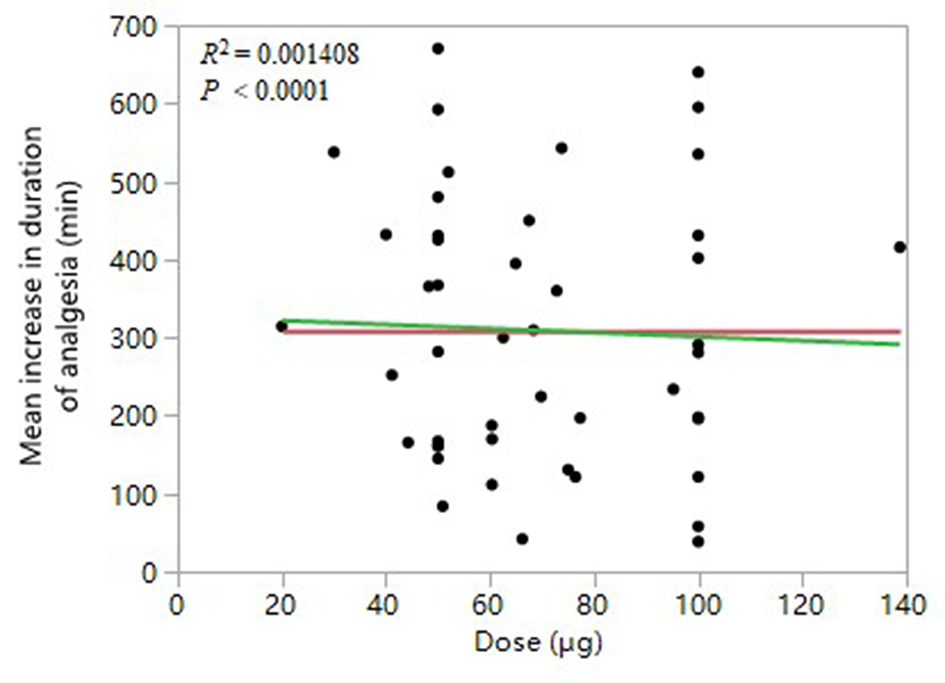


**Additional file 3: Figure S2** Regression analysis of perineural DEX dose and mean increase in DOA when combined with long-acting LAs (pink line: mean line; green line: fitting line). Abbreviations: DEX, dexmedetomidine; DOA, duration of analgesia; LA, local anesthetic
