# Supplementary material for: Optimal dose of perineural dexmedetomidine to prolong analgesia after brachial plexus blockade: a systematic review and Meta-analysis of 57 randomized clinical trials
Source: BMC Anesthesiol. 2021 Sep 28;21:233. doi: 10.1186/s12871-021-01452-0 (PMC8477554; doi:10.1186/s12871-021-01452-0)
Supplement: Supplementary file 4 — Additional file 4. Regression analysis of perineural DEX dose and mean increase in DOA when combined with short−/intermediate-acting LAs (pink line: mean line; green line: fitting line). Abbreviations: DEX, dexmedetomidine; DOA, duration of analgesia; LA, local anesthetic. [file 12871_2021_1452_MOESM4_ESM.docx]

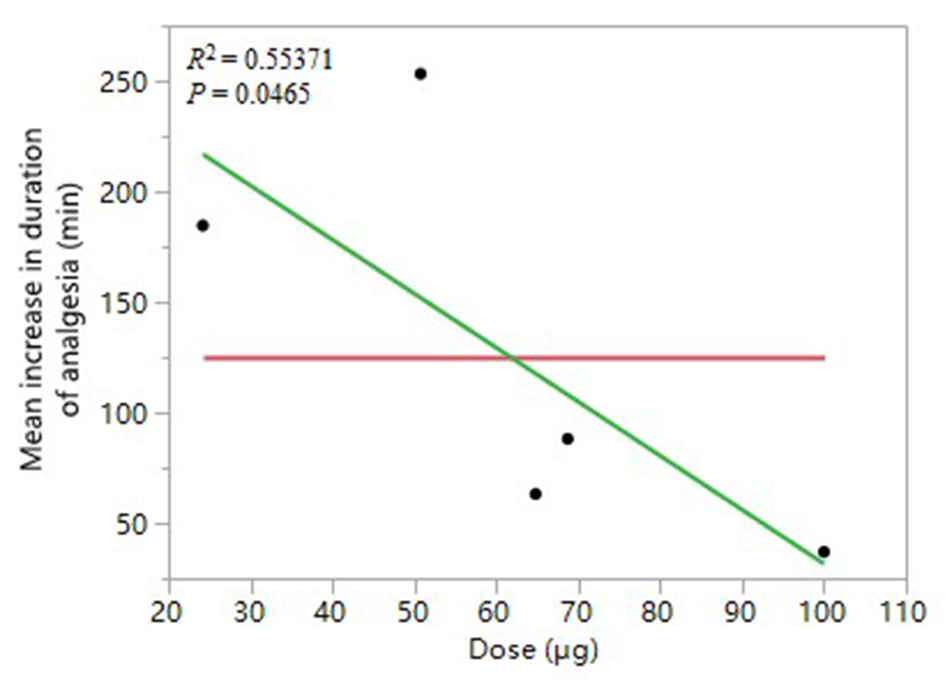


**Additional file 4: Figure S3** Regression analysis of perineural DEX dose and mean increase in DOA when combined with short-/intermediate-acting LAs (pink line: mean line; green line: fitting line). Abbreviations: DEX, dexmedetomidine; DOA, duration of analgesia; LA, local anesthetic
