# Supplementary material for: Optimal dose of perineural dexmedetomidine to prolong analgesia after brachial plexus blockade: a systematic review and Meta-analysis of 57 randomized clinical trials
Source: BMC Anesthesiol. 2021 Sep 28;21:233. doi: 10.1186/s12871-021-01452-0 (PMC8477554; doi:10.1186/s12871-021-01452-0)
Supplement: Supplementary file 6 — Additional file 6. Effect of perineural DEX by dose administered (≤60 μg or>60 μg) on hypotension. Abbreviations: DEX, dexmedetomidine; LA, local anesthetic; CI, confidence interval. [file 12871_2021_1452_MOESM6_ESM.docx]

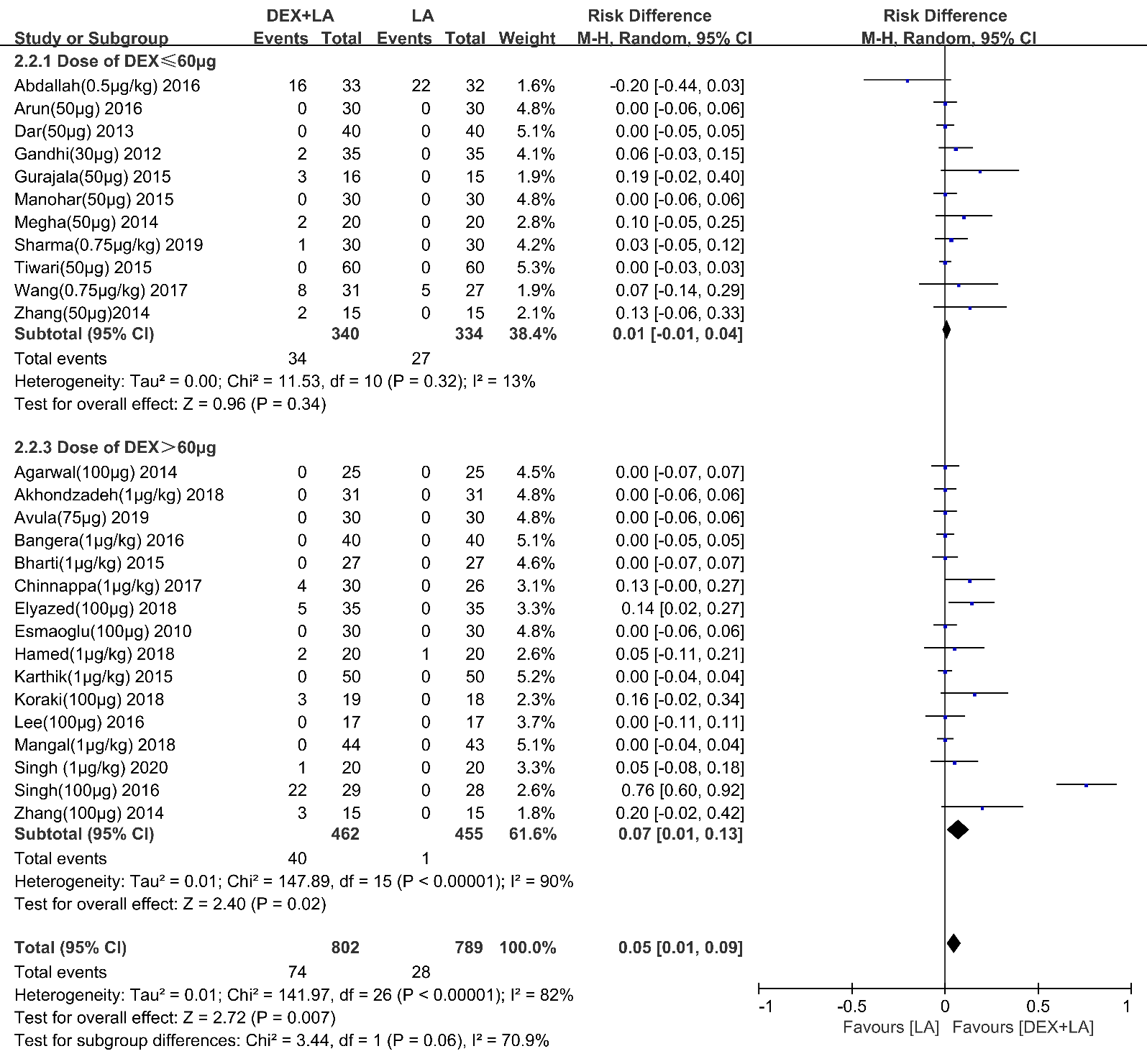


**Additional file 6: Figure S5** Effect of perineural DEX by dose administered (≤60μg or＞60μg) on hypotension. Abbreviations: DEX, dexmedetomidine; LA, local anesthetic; CI, confidence interval
